# Parvovirus B19‐associated graft failure after allogeneic hematopoietic stem cell transplantation

**DOI:** 10.1002/cnr2.1403

**Published:** 2021-05-01

**Authors:** Nabila Rattani, Christina Matheny, Michael J. Eckrich, Lisa M. Madden, Troy C. Quigg

**Affiliations:** ^1^ Pediatric Blood and Marrow Transplantation Program Texas Transplant Institute, Methodist Children's Hospital San Antonio Texas USA; ^2^ Present address: Pediatric Blood and Marrow Transplantation Program, Helen DeVos Children's Hospital, 10th Floor MC185, 100 Michigan St. NE Grand Rapids Michigan MI 49503. USA

**Keywords:** allogeneic hematopoietic stem cell transplantation, graft failure, parvovirus B19 infection

## Abstract

**Background:**

Parvovirus B19 (PVB19) infection has been implicated in allograft failure or dysfunction in solid organ transplantation (SOT) and allogeneic hematopoietic stem cell transplantation (allo‐HSCT), but the literature is limited.

**Case:**

Two pediatric patients were diagnosed with PVB19 infection around the time of allo‐HSCT graft failure. Both cases were secondary graft failure and required second allo‐HSCT.

**Conclusion:**

There are many risk factors and potential confounders in determining the exact etiology of graft failure after allo‐HSCT. These two cases highlight the importance of including PVB19 in the diagnostic evaluation for graft failure. PVB19 infection may be an important risk factor for allo‐HSCT graft failure.

## 1 INTRODUCTION

Parvovirus B19 (PVB19) is a single‐stranded DNA virus that commonly infects young children and is self‐limited in immunocompetent individuals with symptoms ranging from characteristic “slapped cheek” rash, viral exanthem, flu‐like symptoms, arthralgias, and occasional pure red cell aplasia.^1^, ^2^ PVB19 has a predilection for erythroid progenitor cells which can result in pure red cell aplasia, but PVB19 has also been associated with transient aplastic crisis.^2^, ^3^, ^4^ The exact mechanism of PVB19‐associated bone marrow failure is not fully understood. Literature suggests that PVB19 has ability to replicate more efficiently in the relatively more hypoxic bone marrow microenvironment, leading to viral‐induced bone marrow suppression.^3^ While mild infection occurs in normal hosts, PVB19 infection in immunocompromised hosts has been associated with hepatitis, pneumonitis, myocarditis, chronic pure red cell aplasia, and allograft dysfunction after solid organ transplant (SOT) and allogeneic hematopoietic stem cell transplantation (allo‐HSCT).^4^, ^5^, ^6^, ^7^, ^8^, ^9^, ^10^, ^11^, ^12^, ^13^, ^14^, ^15^

PVB19‐associated bone marrow graft failure has been rarely reported after allo‐HSCT.^5^, ^12^, ^13^ There are many risk factors and potential confounders in determining the exact etiology of graft failure after allo‐HSCT. Thorough evaluation for the etiology of graft failure is critical to optimize treatment and outcomes for patients. We describe two pediatric cases of PVB19‐associated graft failure after allo‐HSCT, adding to the limited existing literature and highlighting the importance of inclusion of PVB19 in the diagnostic evaluation of graft failure.

## 2 CASE

### 2.1 Patient 1

A 1‐year‐old male with leukocyte adhesion deficiency (LAD) received reduced intensity conditioning followed by an HLA‐matched sibling bone marrow graft (Table 1). He achieved neutrophil and platelet engraftment by days +23 and +25, respectively. Day +29 chimerism was 100% donor unseparated (whole blood by STR analysis). The first signs of allograft dysfunction were at day +60 with reduction in percent donor engraftment (71% donor unseparated, 72% donor CD15+ myeloid, and 97% donor CD3+ lymphoid). He developed biopsy‐proven acute gastrointestinal graft‐versus‐host disease (GVHD) at day +77, responsive to systemic steroids. Evaluation for unexplained and persistent transfusion‐dependent normocytic normochromic anemia at day +88 revealed PVB19 by blood PCR (3900 copies/mL). In retrospect, he had history of intermittent and unexplained, non‐palpable, non‐specific blanching erythematous rash associated with crying (perceived pain) during the early post‐engraftment period. We suspect that the indolent rash and pain he experienced were likely early manifestations of PVB19 infection, which would have preceded the decrease in day +60 donor chimerism studies.

**TABLE 1:** Summary of patient characteristics

	Patient 1	Patient 2
Age at allo‐HSCT (years)	1	13
Sex	Male	Male
Diagnosis	Leukocyte adhesion deficiency, Type 1	AML CR1 (MRD negative)
Conditioning^a^	Reduced intensity	Myeloablative
Conditioning regimen^a^	Alemtuzumab Fludarabine Thiotepa Melphalan	Fludarabine Busulfan
Graft source	HLA‐Matched Sibling Bone Marrow	Haploidentical Sibling Bone Marrow
ABO compatible	Yes	Yes
Graft cell doses^a^	4.85 × 10^8^ TNC/kg 9.74 × 10^6^ CD34+/kg	3.6 × 10^8^ TNC/kg 4.51 × 10^6^ CD34+/kg
GVHD prophylaxis^a^	Tacrolimus Methotrexate	Post‐transplant cyclophosphamide Tacrolimus Mycophenolate mofetil
PVB19 blood PCR at diagnosis (copies/mL)	3900 (Day +88)	<500 (Day +40)
PVB19 bone marrow PCR	Not Done	Detected
GVHD and treatment^a^	Acute GI GVHD Methylprednisolone	Acute GI GVHD Methylprednisolone
Transfusion‐dependent anemia at time of PVB19 diagnosis	Yes	Yes
Lowest Hgb level, (mg/dL)	6.7	6.5
Leukopenia at time of PVB19 diagnosis	No (Autologous Recovery)	Yes (Aplastic)
Lowest WBC count at time of PVB19 diagnosis (×10^9^ cells/L)	5.5	<0.1
Thrombocytopenia at time of PVB19 diagnosis	Yes	Yes

*Note*: Allo‐HSCT, allogeneic hematopoietic stem cell transplantation; AML, acute myeloid leukemia; CR1, first complete remission; GI, gastrointestinal; GVHD, graft‐versus‐host disease; Hgb, hemoglobin; HLA, Human Leukocyte Antigen; MRD, minimal residual disease; PCR, polymerase chain reaction; PVB19, parvovirus B19; TNC, total nucleated cells; WBC, white blood cell.

^a^ First allo‐HSCT descriptions or events.

The patient received intravenous immunoglobulin (IVIG) replacement at 0.5 g/kg monthly for IgG levels <400 mg/dL per routine post‐HSCT monitoring and care concurrent with detection of the PVB19 viremia (two doses administered). His IVIG replacement therapy overlapped the same time period that the PVB19 infection was recognized and subsequently cleared. Follow‐up PVB19 blood PCR was negative at day +102. At day +105, repeat blood chimerism studies decreased to 9% unseparated and 5% myeloid donor engraftment. Flow cytometry revealed repopulation of original LAD phenotype, confirming secondary graft failure. The patient had autologous immune recovery and his blood PVB19 PCRs remained negative. He received a second allo‐HSCT using myeloablative busulfan and cyclophosphamide followed by G‐CSF mobilized peripheral blood stem cells from the same donor at day +225 from his first allo‐HSCT. At the time of this report, Patient 1 is 23 months status post his second allo‐HSCT with no evidence of GVHD or underlying LAD. He remains 100% donor by unseparated, myeloid, and lymphoid chimerism. He has had no recurrence of PVB19 viremia following his second allo‐HSCT.

### 2.2 Patient 2

A 13‐year‐old male with high‐risk acute myeloid leukemia (AML) received myeloablative conditioning followed by a haploidentical sibling bone marrow graft (Table 1). He achieved neutrophil engraftment by day +16, but never achieved platelet engraftment. Patient 2 had a non‐specific blanching rash with bone pain during the peri‐engraftment period. He developed cytomegalovirus (CMV) viremia and was treated and resolved with ganciclovir and valganciclovir early post‐HSCT. Day +26 chimerism was 89% donor unseparated and 100% donor myeloid. Bone marrow exam on day +40 showed mixed chimerism, no recurrent AML, but detectable PVB19 by qualitative PCR. Blood PVB19 PCR on day +40 was positive (<500 copies/mL). IVIG 1 g/kg was administered for empiric treatment on day +43. The patient was diagnosed with secondary graft failure with persistent severe transfusion‐dependent pancytopenia. Repeat blood PVB19 PCRs remained positive at days +52 and +61 (312 and 384 copies/mL, respectively). IVIG 1 g/kg was repeated on day +61. Given the severity of his pancytopenia and associated high risk for other opportunistic infections, we rapidly proceeded with a second allo‐HSCT. Blood PVB19 PCR at day +69 (day prior to second allo‐HSCT) remained positive (322 copies/mL).

He received a rescue allo‐HSCT from the same donor with G‐CSF mobilized peripheral blood stem cells following nonmyeloablative conditioning with fludarabine, cyclophosphamide and 400 cGy TBI. Patient 2 achieved neutrophil and platelet engraftment after second allo‐HSCT at days +15 and + 33, respectively, with 100% donor engraftment by unseparated and lineage‐specific chimerism analysis. His second allo‐HSCT course was complicated by acute gastrointestinal GVHD that resolved with low dose systemic steroids (0.5 mg/kg/day) and extracorporeal photopheresis (ECP), as steroid‐sparing therapy. PVB19 blood PCRs remained positive at low levels until a single negative PCR resulted at day +54 after second allo‐HSCT. At day +68, his blood PVB19 PCR was again detected (<199 copies/mL) and persisted at this level until day +166 when viral loads began to increase (96 400 copies/mL). By day +174 after second allo‐HSCT, he was readmitted with unexplained fever and found to have a PVB19 PCR viral load of 1.38 × 10^10^ copies/mL. He was given IVIG 1 g/kg daily × 3 consecutive days, fevers resolved, and was discharged home. PVB19 viral loads began to decline the week following IVIG treatment (2.4 million copies/mL) and the patient was maintained on monthly IVIG 0.5 g/kg until he had evidence of T‐ and B‐cell immune reconstitution 8 months later. Over a course of 12 months, his blood PVB19 PCRs declined to a low level of detection (<199 copies/mL) but were never negative. With documented immune recovery, response to vaccinations, and no evidence of residual cytopenias or graft dysfunction, blood PVB19 PCR surveillance was stopped. At the time of this report, Patient 2 is 26 months status post his second allo‐HSCT, without evidence of recurrent AML or chronic GVHD, and remains 100% donor across all chimerism studies.

## 3 DISCUSSION

PVB19 infection is a rarely reported complication following SOT or allo‐HSCT. Cases of allograft loss or dysfunction have been reported following SOT and allo‐HSCT, but the literature is limited. The two cases described in this case report add to existing rare reports of PVB19‐associated graft failure after allo‐HSCT.^5^, ^12^, ^13^

The first report of PVB19 infection after renal transplantation was published in 1986.^7^ PVB19 infection was implicated in a series reporting five pediatric SOT recipients who developed anemia and reticulocytopenia.^11^ Significantly, PVB19 infection has been associated with allograft loss or dysfunction after SOT.^5^, ^8^, ^9^, ^10^, ^11^, ^12^, ^13^, ^14^

Rare cases of PVB19 infection during allo‐HSCT have been reported in the literature. One case described a 14‐year‐old girl with acute promyelocytic leukemia who had abrupt onset pure red cell aplasia induced by PVB19 after an HLA‐matched bone marrow transplant.^12^ In another case report, a 38‐year‐old male was admitted with severe pancytopenia and multi‐organ failure with PVB19 infection that occurred 1 year post T‐cell depleted bone marrow transplantation from a matched unrelated donor for refractory chronic lymphocytic leukemia.^13^ Such cases demonstrate that PVB19 infection can lead to more severe disease in allo‐HSCT recipients.

Allograft loss or dysfunction can be a devastating and life‐threatening complication of SOT or allo‐HSCT. In a systematic review of the literature summarizing cases of PVB19 infection after SOT (n = 74) and allo‐HSCT (n = 24), allograft loss or dysfunction occurred in 10.4% of patients.^5^ The majority of PVB19 (70%) occurred in the first 6 months after transplantation. Co‐infection with CMV and human herpesvirus 6 were found in 1% of cases, respectively. While the authors confirmed that serological tests are insufficient for diagnosis of PVB19 infection in immunocompromised patients (29% were negative), 96% were positive by PCR.

We report two cases of diagnosed PVB19 infection and secondary graft failure after allo‐HSCT. Recognition of PVB19 infection as a possible risk factor for graft failure is important for diagnostic consideration. Anemia in the early post‐HSCT period is expected and unless other symptoms or complications develop, PVB19 is rarely considered. Allo‐HSCT patients may not manifest with typical rash and joint pain due to an inadequate immune response or when symptoms are present, those symptoms are often attributed to donor cell engraftment. One report described two cases of erythematous rash in two PVB19 positive immunocompromised patients early after allo‐HSCT.^15^ Both patients described in this case report had similar histories of non‐specific rash and pain in the post‐engraftment period that may have been indolent manifestations of PVB19 infection. Persistent hypoproliferative transfusion‐dependent anemia led to PVB19 evaluation and diagnosis in patient 1 and preceded secondary graft failure. Patient 1's clinical course led our team to test for PVB19 infection in patient 2 who suffered earlier and life‐threatening secondary graft failure with resultant aplastic anemia.

Recipients of SOT and allo‐HSCT are at risk for PVB19 infection due to poor immune globulin production and T‐cell function during the post‐transplant period and while on immune‐suppressing therapies. Intravenous immune globulin (IVIG) is considered the only treatment option for PVB19 infection. IVIG treatment targets associated anemia, not fulminant graft failure or dysfunction, and there is significant risk for relapse of anemia.^5^, ^11^, ^13^ High‐dose IVIG is a source of neutralizing autoantibodies as the majority of the adult population has anti‐B19 antibodies and the treatment effects are evidenced by rise in reticulocyte count and corresponding rise in the hemoglobin level.^4^ IVIG has also been utilized to treat chronic pure red cell aplasia caused by persistent PVB19 infection in HIV‐infected patients.^16^, ^17^ Both patients described in this report received IVIG around the time of initial PVB19 infections. Patient 1 was receiving replacement IVIG (0.5 g/kg) for acquired hypogammaglobulinemia concurrent with the timeline of his PVB19 infection. The secondary graft failure ensued in Patient 1, but the patient had autologous blood count and immune recovery. IVIG was administered at higher doses earlier in the diagnosis of graft failure in Patient 2, but there was no appreciable improvement in his pancytopenia leading to urgent rescue allo‐HSCT. When PVB19 viremia recurred after Patient 2's second allo‐HSCT, we administered high‐dose IVIG comparable to reports in HIV‐treated patients.^16^, ^17^ As discussed, fevers resolved, viral loads improved, and there were no concerns for graft loss. Based on the observations in this report, we hypothesize that primary PVB19 infection at earlier timepoints after allo‐HSCT may be more impactful on graft failure or dysfunction than reactivations or infections that occur later in the post‐HSCT period. However, additional data are needed to elucidate the mechanisms and associated kinetics of PVB19‐associated graft failure after allo‐HSCT.

There are many potential risk factors and confounders for graft failure after allo‐HSCT such as underlying disease, type of conditioning, graft source and infused cell doses, infections (pre‐ and post‐allo‐HSCT), and drug toxicities. These potential confounders could limit the association of PVB19 infection and graft failure. Patient 1 in our report received reduced intensity conditioning but had no other identifiable risk factors for graft failure. Patient 2 received a haploidentical graft, had low level CMV viremia, and received ganciclovir and valganciclovir in the early post‐engraftment period; all potential contributors to graft failure. Although we cannot exclude the potential impact of CMV viremia and drug‐induced toxicity in Patient 2 on graft failure, he received his second allo‐HSCT from the same sibling haploidentical donor and engrafted without incident. The only unifying feature of graft failure between our two cases was PVB19 infection occurring early after first allo‐HSCT.

In this report, we describe two cases of PBV19 infection associated with secondary graft failure after allo‐HSCT. These cases add to the limited existing literature and highlight the importance of including PVB19 infection in the diagnostic evaluation for graft failure after allo‐HSCT. Quantitative PCR for PVB19 should be incorporated into comprehensive infectious and immunological testing to evaluate etiology of graft failure after allo‐HSCT. IVIG may be beneficial in the management of patients with PVB19 infection after allo‐HSCT but is unlikely to reverse fulminant graft failure occurring early after allo‐HSCT.

## CONFLICT OF INTEREST

The authors have stated explicitly that there are no conflicts of interest in connection with this article.

## AUTHOR CONTRIBUTIONS

**Nabila Rattani:** Conceptualization; writing‐original draft; writing‐review & editing. **Christina Matheny:** Conceptualization; writing‐original draft; writing‐review & editing. **Michael Eckrich:** Conceptualization; writing‐original draft; writing‐review & editing. **Lisa Madden:** Conceptualization; writing‐original draft; writing‐review & editing. **Troy Quigg:** Conceptualization; supervision; writing‐original draft; writing‐review & editing.

## ETHICAL STATEMENT

No institutional review board approval was required as retrospective review of two patient cases for publication is not considered human subjects' research consistent with US Federal Policy for the Protection of Human Subjects. Therefore, no patient consent was required.

## Data Availability

The data that support the findings of this study are available on request from the corresponding author. The data are not publicly available due to privacy or ethical restrictions.
